# Improving case fatality ratio estimates in ongoing pandemics through case-to-death time distribution analysis

**DOI:** 10.1038/s41598-025-89441-y

**Published:** 2025-02-13

**Authors:** Zia Farooq, Henrik Sjödin, Joacim Rocklöv, Åke Brännström

**Affiliations:** 1 Department of Epidemiology and Global Health, Umeå University, Umeå, 901 87 Sweden; 2 Heidelberg institute of global health and Interdisciplinary centre for scientific computing, University of Heidelberg, Im Neuenheimer Feld 205, 69120 Heidelberg, Germany; 3 Department of Mathematics and Mathematical Statistics, Umeå University, Umeå, 901 87 Sweden; 4 Complexity Science and Evolution Unit, Okinawa Institute of Science and Technology Graduate University, Kunigami, Japan

**Keywords:** Case fatality ratio, CFR, COVID-19, Case-to-death times, Distributed-delay method, Particle-swarm optimization, Statistics, Infectious diseases

## Abstract

The case fatality ratio (CFR) is a vital metric for assessing the disease severity of novel pathogens. The widely used direct method of CFR estimation—the ratio of total confirmed deaths to total confirmed cases—is inherently simplistic, as it fails to account for the essential time lag between case confirmation to death, and reporting delays. These limitations often lead to biased CFR estimates, particularly in the early stages of outbreaks. This study introduces a novel approach—the distributed-delay method that, like the direct method, utilizes publicly available aggregate time-series data on cases and deaths. It estimates CFR by flexibly incorporating a case-to-death time distribution without requiring a priori assumptions on distribution parameters. Using a fitting approach to forecast case fatalities based on known or assumed case-to-death time distributions, the method consistently recovers true CFR much earlier than the direct method under various simulation settings. These settings reflect variability in disease severity, uncertainties in case-to-death time parameters, and limited knowledge of case-to-death time distributions. It outperforms other methods such as Baud’s, which assumes a non-zero constant case-to-death time, and the Generalized Baud’s method, which allows for a direct comparison with our new approach. While evaluations based on empirical data are challenging, our conclusions are supported by CFR estimates obtained using empirical COVID-19 data from 34 countries. As an added value, this analysis also demonstrates a significant negative association between eventual CFR and the expected case-to-death time within the context of COVID-19 data. Our study highlights the complexities of inferring real-time CFR from aggregate time-series case and death data, highlighting that refining this method can lead to accurate real-time CFR estimations for actual outbreaks.

**Supplementary Information:**

The online version contains supplementary material available at 10.1038/s41598-025-89441-y.

## Introduction

Accurate disease severity estimation is crucial during disease outbreaks to aid informed decisions about risk assessment and outbreak response actions^1–3^. The case fatality ratio (CFR) is a commonly used quantification of disease severity to provide the proportion of deaths attributed to a given disease among the number of confirmed cases^4^. However, this quantity often becomes clear only sometime after an outbreak when data on the number of disease cases and case fatalities becomes available. The most widely used quantification method, which we refer to as the *direct method*, for estimating crude estimates of CFR is given simply by the ratio of cumulative fatalities divided by the cumulative cases up to a given day. However, it is well established that the direct method can massively underestimate the true CFR during an ongoing outbreak^5^. This is because actual delays between case registrations and case fatalities do not harmonize with the method’s underlying assumption of zero time from case registration to death, resulting in biased disease severity estimates, particularly during an outbreak^6,7^. The problem becomes particularly acute for diseases that exhibit longer delays between disease infection and following death^8^.

During the COVID-19 pandemic, the direct method was an extensively used estimator of the CFR. There have been several studies on the CFR of COVID-19 ^8–15^, and some have attempted to improve the CFR estimation^8,9,13–15^. In one of the studies, Baud et al. (2020) tried to resolve this problem by accounting for a non-zero case-to-death time, where they assume specifically a fixed 14-day case-to-death time, without variation around that mean^9^. Baud’s method, as previously reported^16^ and we will illustrate here, exhibits tangible deviations from true CFR early during an outbreak.

While Baud et al.’s (2020) method is a relatively straightforward alternative to the direct method, one must also recognize two limitations: (1) The mean case-to-death time needs to be known or estimated by other means, and (2) the variation around this mean is not zero and the case-to-death times actually follow a probability distribution. The notion of distributed case-to-death times is arguably vital for CFR estimation, as it allows probabilistic predictions of future fatalities from present cases. Even if we have information on the number of cases at a given point early in an outbreak, we can only obtain the CFR if we know the fraction of those cases that have or will result in a fatality.

Here, we present a new quantitative method that circumvents these two limitations, with the only requirement being the assumption of a functional form describing how case-to-death times are distributed (see Table 1 for comparison). Case-to-death times should, however, be expected to be positively skewed, and it has been shown that lognormal or gamma-distributed case-to-death times are common^17,18^.

In short, the method acknowledges variable case-to-death times where distribution parameters are emergent by minimizing residuals between estimated and actual case fatalities during ongoing outbreaks. We refer to this method as the *distributed-delay method* and demonstrate it through simulations and empirical COVID-19 data. By comparing our method with the direct and Baud’s method, we show how our method addresses the key limitations of these simpler methods using publicly available cases and deaths. The method typically generates improved CFR estimates much earlier than these methods and provides interesting insights into the association between the CFR and expected case-to-death times in empirical data settings.

**Table 1:** A comparison of different methods for estimating case fatality ratios with the distributed-delay method.

Quantification method	Mean case-to-death time	Dispersion around the mean	Method brief description	A priori information (data)	When during an outbreak, it provides reliable estimates?
Direct	Constant, 0	0	Reported deaths to cases ratio	Population-level cases and fatalities	When the outbreak wanes
Ghani^5^	Average of empirical case-to-death time	Dispersion in empirical case-to-death time	Kaplan-Meier	Individualized recoveries and fatalities data	Subject to sufficient data availability/post exponential growth phase
Jewell^19^	Average of empirical case-to-death time	Dispersion in empirical case-to-death time	Kaplan-Meier	Individualized fatalities and recoveries data	Subject to sufficient data availability/post the exponential-growth phase
Nishiura^17^	Mean based on other previous outbreaks	Dispersion based on other previous outbreaks	Exponential/gamma distribution with maximum likelihood	Distribution parameters and population-level cases and fatalities	Early in an ongoing epidemic subject to the availability of assumed distribution parameters
Thomas^15^	Fitted to population- level case fatalities	0	Time-shifted distribution	Population-level cases and fatalities	Post the exponential-growth phase
Baud’s^9^	14 days	0	Constant case-to-death time	Population-level cases and fatalities	Post the exponential-growth phase subject to the availability of case-to-death time of disease under consideration
Generalized Baud’s (this study)	Fitted to population-level case fatalities	0	Delta distribution of case-to-death time	Population-level cases and fatalities	Post the exponential-growth phase
Distributed-delay (this study)	Fitted to population- level case fatalities	Fitted to population-level case fatalities	Lognormal/gamma distribution	Population-level cases and fatalities	In an ongoing epidemic/pandemic/outbreak

## Methods

The case fatality ratio (CFR) is often regarded as an ambiguous term in the literature due to its varying definitions related to what constitutes a ‘case’^8,17,20^. In this study, we define CFR as the ratio of deaths to confirmed cases alone. The denominator could also encompass the total number of infections, which would then appropriately be termed the infection fatality rate (IFR). Nonetheless, identifying all infected individuals is practically infeasible in the early phase of a novel outbreak, so the denominator is, in practice, often restricted to diagnosed cases, which is often a more applicable measure. While the method could easily be applied to data on all infected, would such data be available? The instance of the method we present here is based on confirmed case data and, therefore, restricted to considerations on CFR.

### Overview of the distributed-delay method

The fundamental idea of the distributed-delay method is to capture the essential processes that govern the case fatality ratio of a given outbreak.

**Fig. 1:**
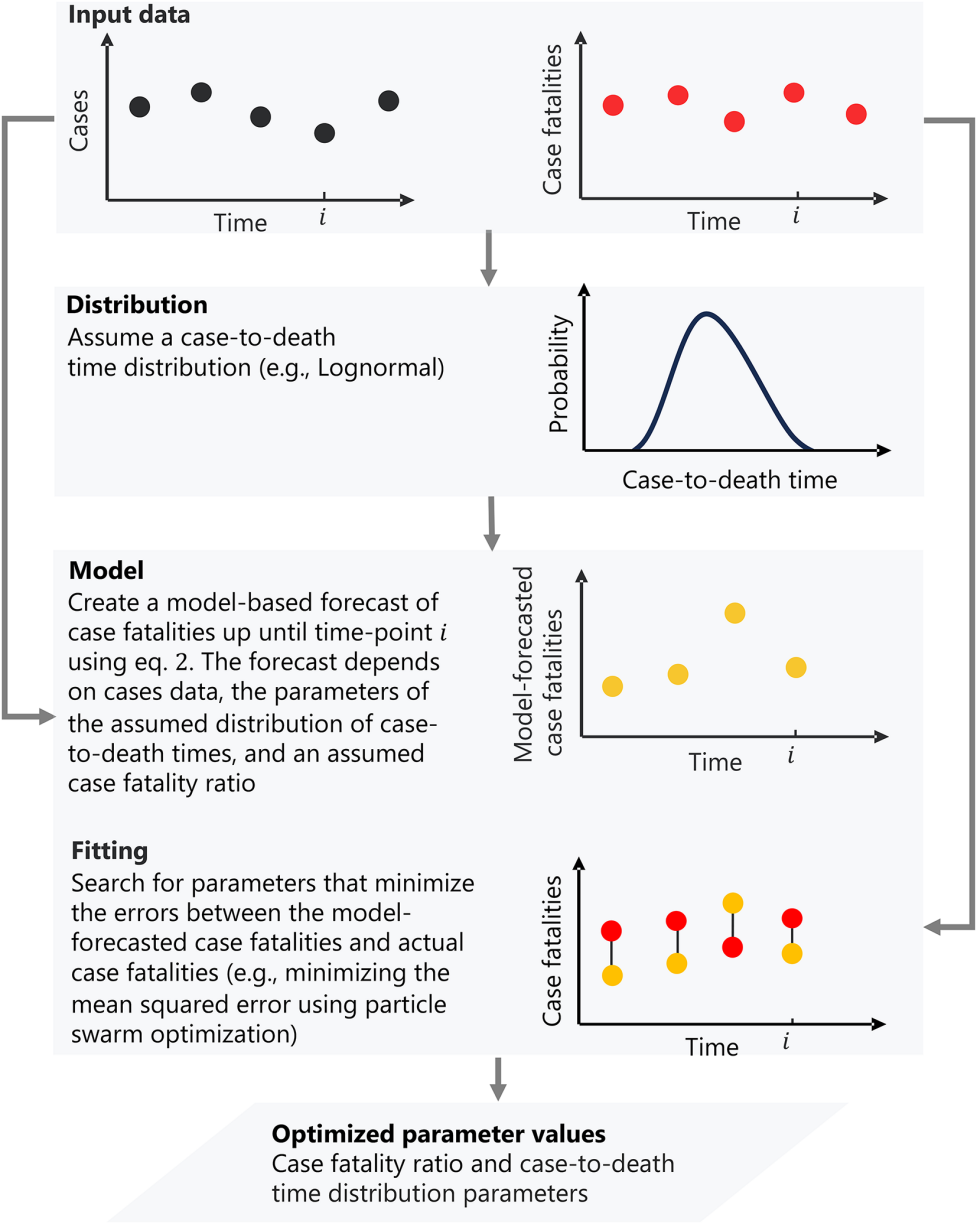
Conceptual framework of the distributed-delay method. In the first step, we assume a probability distribution $\:p\left(t,\:\varTheta\right)$, for the time from case registration to death registration with parameters, Θ. Using this distribution and an assumed case fatality ratio $\lambda$, we forecast case fatalities each day up until time point $i$ since the start of the outbreak/epidemic. The forecast is created using the model (Eq.2). We then search for the parameters that give the best fit between actual and model-based fatalities through minimization of the error (here, minimizing the squared error using particle swarm optimization, PSO). This gives the estimated case fatality ratio $\lambda\:$and the estimated distribution parameters Θ.

The key problem is to account for the fact that there is some time between case registration and an eventual fatality. This case-to-death time varies between cases, diseases, and populations depending on, for instance, its age distribution. This is resolved by correctly accounting for a distribution of case-to-death times when quantifying the CFR (Fig. 1). Using this distribution and an assumed CFR value (denoted by $\lambda$), we can forecast case fatalities corresponding to that CFR each day until the given time-point $i$, described by equation (2) in the next section.

An applicable CFR quantification method should ideally also base estimations on information from the very outbreak for which we estimate the CFR and not depend on earlier outbreak data. Estimation of the CFR and distribution parameters of an outbreak is obtained by fitting model-based case fatalities (i.e., Eq. (2)) to actual or reported daily case fatalities data by minimizing residuals between the two using appropriate optimization methods on the outbreak (Fig. 1) and in that process, the correct CFR value for that outbreak data is estimated.

These are the two fundamental principles of the distributed-delay method.

### Modelling case fatalities

We assume that the case-to-death times $t$ for cases for which the disease proved fatal, follow some probability distribution given by the probability density function $\:p\left(t,\:\varTheta\right)\:$where $\varTheta\:$ denotes its parametric space. The probability that an individual has died from the disease within $t$ days after it was reported as a case is then given by the cumulative probability density function

1 $$\:P\left(t,\varTheta\right)={\int\:}_{0}^{t}\:p\left(x,\:\varTheta\right)\text{d}x,\:\:\:t>0\:,$$

such that the number of case fatalities $\:{{(F}_{D}}_{i}$) until the day $\:i$ since the start of the outbreak is given by

2 $$\:{{F}_{D}}_{i}\left(\lambda\:,\varTheta\right)=\lambda\:\sum\:_{t=1}^{i}P\left(t,\varTheta\right){C}_{i-t+1},\:\:$$

where ${C}_{i-t+1}$ denotes the number of reported new cases at day $i-t+1$ since the start of the outbreak.

### Estimating the correct CFR and distribution parameters by optimization

To estimate CFR and case-to-death time distribution parameters, the modeled case fatalities (i.e., Eqs. (1)-(2) ) are fitted to case fatality data by finding the minimum of the sum of squared differences between modeled case fatalities and case fatality data (i.e., simulated or empirical; see Results section). We use global optimization by applying a particle swarm optimization (PSO) algorithm (see section: Optimization and parameter estimation for details)^21^. This numerical fitting approach requires the case fatality model ${F}_{{D}_{i}}\left(\lambda\:,{\Theta}\right)$ to be fully parameterized.

### Empirical COVID-19 data and generation of simulated data

We evaluated the distributed-delay method on simulations-generated data (see Table 2) and empirical COVID-19 data.

We extracted empirical COVID-19 data on daily cases and case fatalities in 34 countries from online sources (see Supplementary: Table S1 for countries names)^22^.

To eliminate any noise in studying the performance of the distributed-delay method, we also provide a controlled setting with complete information on the underlying processes of case fatalities. By generating simulated case-fatality data based on empirical COVID-19 cases data from the spring 2020 COVID-19 outbreak in South Korea^18^, we could control four factors: (1) true CFR, (2) true mean and (3) standard deviation of case-to-death times, and (4) true functional form of case-to-death time distribution (e.g., lognormal or gamma).

To generate simulated case fatalities data that satisfy a given case fatality ratio $\lambda$, we draw for each day and reported case a random number $r$ between 0 and 1 and assume that the reported cases would not survive the disease for $r<\lambda$, or else survive the disease. We draw case-to-death time for each of the fatal cases from an assumed time-continuous case-to-death time distributions, i.e., with $P\left(t,{\Theta}\right):= P\left(t,m,s\right)$ equal to lognormal or gamma with a given mean $m$ and standard deviation $s$ of case-to-death times (see Results section). Since we use a daily temporal resolution, the drawn continuous case-to-death times are rounded to nearest integers. Table 2 provides details on parameterization of 14 simulation scenarios across which variation in the four factors are evaluated.

**Table 2:** Summary of assumed parameters, the simulation and the fitting distributions used to analyze the sensitivity and robustness of distributed-delay method.

No	Case-to-death time distribution	Mean (m) [a, b]	Standard deviation (s) [a, b]	CFR ($\:\varvec{\lambda\:}$) (%)	Fitting distribution	Reference Figure: Panel
1	Lognormal	8.6^*^ [0.1, 30]	6.7^*^ [0.1, 30]	**2%**	Lognormal	3 A, 4 A
2	Lognormal	8.6^*^ [0.1, 30]	6.7^*^ [0.1, 30]	**5%**	Lognormal	3 A, 4 A
3	Lognormal	8.6^*^ [0.1, 30]	6.7^*^ [0.1, 30]	**10%**	Lognormal	3 A, 4 A
4	Lognormal	8.6^*^ [0.1, 30]	6.7^*^ [0.1, 30]	**15%**	Lognormal	3 A, 4 A
6	Lognormal	**14** [0.1, 40]	6.7^*^ [0.1, 30]	10%	Lognormal	3B, 4B
7	Lognormal	**21** [0.1 50]	6.7^*^ [0.1, 30]	10%	Lognormal	3B, 4B
9	Lognormal	8.6^*^ [0.1, 30]	14 [0.1, 40]	10%	Lognormal	3 C, 4 C
10	Lognormal	8.6^*^ [0.1, 30]	21 [0.1, 50]	10%	Lognormal	3 C, 4 C
11	**Gamma**	8.8^*^ [0.1, 30]	5.7^*^ [0.1, 30]	10%	**Lognormal**	3D, 4D
12	**Lognormal**	8.6^*^ [0.1, 30]	6.7^*^ [0.1, 30]	10%	**Gamma**	3D, 4D
13	**Perturbed** **Lognormal**	8.6^*^+**Uniform[-7**,**7]** [0.1, 30]	6.7^*^ [0.1, 30]	10%	Lognormal	3D, 4D
14	**Gamma**	8.8^*^ [0.1, 30]	5.7 [0.1, 30]	10%	Gamma	3D, 4D

The bold-faced text shows the varying parameters/factor in each scenario. [a, b] refers to the lower and upper bound of parametric search space for the Particle-Swarm optimization (PSO) algorithm. *Taken from Linton et al.^18^.

### Unifying case fatality ratio quantification methods under a generalized perspective

The direct method and Baud’s method do not estimate CFR through fitting. To compare them with the distributed-delay method, we standardize their evaluation by assigning comparable properties. This is achievable for the direct and Baud et al. methods^9^, both recovered as special cases in equation (2) when $\:p\left(t,{\Theta}\right)$ assumes a Dirac delta distribution with mean case-to-death time zero or 14 days. Although the direct and Baud’s methods become identical in the sense that they are both defined by a delta distribution, we refer to this special case as Generalized Baud’s method, which we use to fit case-to-death times.

### Case-to-death time distributions

In our analysis, we considered three instances of assumed case-to-death times distributions: (i) lognormal distribution (ii) gamma distribution, and (iii) delta distribution. Below we expand the details on each of the distribution.

Firstly, we used a lognormal distribution $\:p\left(t,{\Theta}\right)\:$of case-to-death time (Eq. 1) with mean $\:\mu$, and standard deviation $\:\sigma$, with days as a temporal unit^18^. We write the lognormal distribution of case-to-death times given in Eq. (3)

3 $$\:p\left(t,{\Theta}\right)=\:p\left(t,\mu\:,\sigma\:\right)=\frac{1}{t\sigma\:\sqrt{2\pi\:}}\text{exp}\left(-\frac{{\left(\text{log}t-\mu\:\right)}^{2}}{2{\sigma\:}^{2}}\right),\:t>0$$

where the parameters $\:\mu\:$, and $\:\sigma\:$, are computed using the transformation of mean (*m*) and standard deviation (*s*) of lognormal random variable as

4 $$\:\mu\:=\text{log}\left(\frac{{m}^{2}}{\sqrt{{s}^{2}+{m}^{2}}}\right),$$

5 $$\:\sigma\:=\sqrt{\text{l}\text{o}\text{g}\left(\frac{s}{{m}^{2}}+1\right)},$$

Next, we also implemented the distributed-delay method with a gamma distribution of case-to-death times with parameters shape ($\:k$) and scale ($\:\theta\:$) given in Eq. (6) as

6 $$\:p\left(t,{\Theta}\right)=\:p\left(t,k,\theta\right)=\frac{1}{{\Gamma}\left(k\right){\theta\:}^{k}}{t}^{k-1}{e}^{-\frac{t}{\theta\:}},\:t>0$$

where the parameters $\:k$, and $\:\theta$, are computed using the transformation of the mean (*m*) and standard deviation (*s*) of gamma random variable as

7 $$\:k=\frac{{m}^{2}}{{s}^{2}},$$

8 $$\:\theta\:=\frac{{s}^{2}}{m},$$

We further implemented the method by assuming a delta distribution of case-to-death time for which the Eq. (1) is replaced by Eq. (9) ( see section “Generalized Baud’s method”)

9 $$\:P\left(t,\mu\right)=\delta\:\left(t|m\right),\:\:\:t>0\:$$

### Optimization procedure for parameters estimation

To estimate the parameters, i.e., case fatality ratio, ${\uplambda}$, and the distribution parameter(s), $\:{\Theta},$ for all the instances of assumed distributions (Eqs. (3)-(9)) we numerically find the combination of these parameters, through a numerical optimization algorithm, that minimizes the residuals between actual and model forecasted case fatalities (Eq. (2)). To apply a numerical optimization algorithm that searches for the combination of parameters that minimizes the residuals, we established an *objective function*$\:f\left({\uplambda}\:,{\Theta}\right)\:$with parametric constraints given by (10)-(11)

10 $$\:f\left(\lambda,\varTheta\right)=\text{a}\text{r}\text{g}\text{m}\text{i}\text{n}\sum\:_{i=1}^{n}{\left({{A}_{D}}_{i}-{{F}_{D}}_{i}\left(\lambda,\varTheta\right)\right)}^{2},\:$$

subject to parametric constraints;

11 $$\:\lambda\:\epsilon\left[0\:1\right],\:\:\varTheta\:\epsilon\left[a\:b\right];\:\:a>b>0,$$

where ${{A}_{D}}_{i}$ and ${{(F}_{D}}_{i})$ are respectively the actual daily and model forecasted case fatalities of $\:n$ days of an outbreak.

Note that in this context, the objective function, $f$, is not in closed form. Consequently, only a numerical solution using a derivative-free optimization algorithm is feasible. We evaluate the problem against various algorithms using the MATLAB R2020a global optimization toolbox^23^. After testing multiple derivative-free optimization algorithms, we find that the metaheuristic particle swarm optimization (PSO) algorithm performs the best^21^.

Unlike gradient-based or simplex methods (e.g., Nelder-Mead^24^), PSO does not rely on assumptions of a smooth or unimodal objective function. Instead, it employs a population-based search that facilitates a broader exploration of the parameter space and minimizes the risk of convergence to local minima. This characteristic was particularly critical given the need to locate the global minimum in our application, where the simplex methods showed limitations in handling the non-smooth and non-convex nature of the problem. Thus, PSO emerged as the most suitable choice for the optimization process.

To apply PSO, constraints on parameters need to be defined for both simulations and empirical data cases. We set these constraints for simulations as provided in Table 2. For empirical COVID-19 estimates we used a lognormal distribution of case-to-deaths times throughout and set constraints for both case-to-death parameters $\:m$ and $\:s$ to $\:a=\:0.1,$ and $\:\:b=\:30$. The distribution parameters derived from the fitting process, are reported in Supplementary Information Table 1.

Here, we illustrate an instance implementation of the lognormal distribution for the optimization algorithm (i.e., Eqs. (1)-(2) together with  (10)-(11). Since the algorithm is computationally intensive (see Supplementary; Computational cost of the distributed-delay method), we first define and then refine a parametric search space. We define a 3-dimensional grid space for parameters where each dimension corresponds to one parameter, i.e., that is, $\:\lambda,\:m$ and $\:s$. To create the 3-dimensional parametric grid, we divide the parametric space of each parameter, subject to the constraints described above, into $\:k$ grid-points. As a result, the number of model-based forecasted case fatalities and fittings to actual fatalities would be on the order of ${k}^{3}$. Here, we choose $\:k=10$, resulting in 1000 fitted instances of parameter sets and corresponding residuals. We then select the parameter sets that provide the minimum and maximum values of residuals. These parameter sets establish the new constraint limits. This enables us to apply the particle swarm optimization algorithm more efficiently with the newly defined parametric constraints and to find the best fit between actual and model-forecasted case fatalities.

One of the critical hyperparameters of PSO for finding the minimum is its *swarm size*—the number of particles in the swarm. Generally, the larger the swarm size, the higher the computational time of the method. To that end, we test multiple swarm sizes (1000, 2500, 5000) and find that a swarm size of 5000 particles results in estimating the optimal parameter values from the distributed-delay method. The parameter *iteration numbers are* also important for the computational time. An insufficiently small number of iterations may bring the search process to an early halt, while extensive iterations could lead to unwarranted computational complexity and extended computational time^25^. In our case, we set this parameter to its default value in MATLAB.

### Generalized Baud’s method

The direct method does not estimate CFR by fitting or Baud’s method. To evaluate the distributed-delay method against these methods should they be fitted to data in the same manner as the direct method, we unify them by accrediting each method’s comparable properties. This is possible for the direct method and the method presented by Baud et al. (2020)^9^, which also are the quantification methods we evaluate against in our results. Both methods are recovered as special cases in Eq. (2) when $\:\text{P}\left(\text{t},{\Theta}\right)$ is assumed to be equal to the Dirac delta distribution (Eq. (9) ) with mean case-to-death time zero (direct method) or 14 days Baud’s method). The delta distribution’s dispersion (e.g., standard deviation) is, by definition, infinitesimal, which is true for both methods since none of them accounts for variation in case-to-death times. We can apply Eq. (2) and obtain case fatalities representing the direct or Baud’s methods.

With this generalization, we can then proceed to estimate CFR and any distribution parameters of the delta distribution, analogous to the distributed-delay method. It follows that the direct methods and Baud’s method converge at this point because the only distribution parameter that can be estimated is the mean case-to-death time since the delta distribution lacks any statistical moment around the mean.

Although the direct method and Baud’s method become identical, we refer to this generalization as the Generalized Baud’s method since it explicitly accounts for non-zero case-to-death times, while the direct method does not.

One could also extend this generalization by allowing for non-zero dispersion, which would recover a method that then assumes normally distributed case-to-death times as the Dirac delta function is obtained in the limit of infinitesimal dispersion in a normal distribution. However, while such a second-order generalization is interesting in and of itself, it would not add to the comparison analyses as it would fall out of scope.

## Results

We compare the results of our method with three other methods: (i) the direct method, which assumes a case-to-death time of zero days; (ii) Baud’s method with a constant 14-day case-to-death time; the ratio of total deaths to the total cases two weeks ago and (iii) Generalized Baud’s method with a free case-to-death time parameter.

### Distributed-delay outperforms on simulated data

**Fig. 2:**
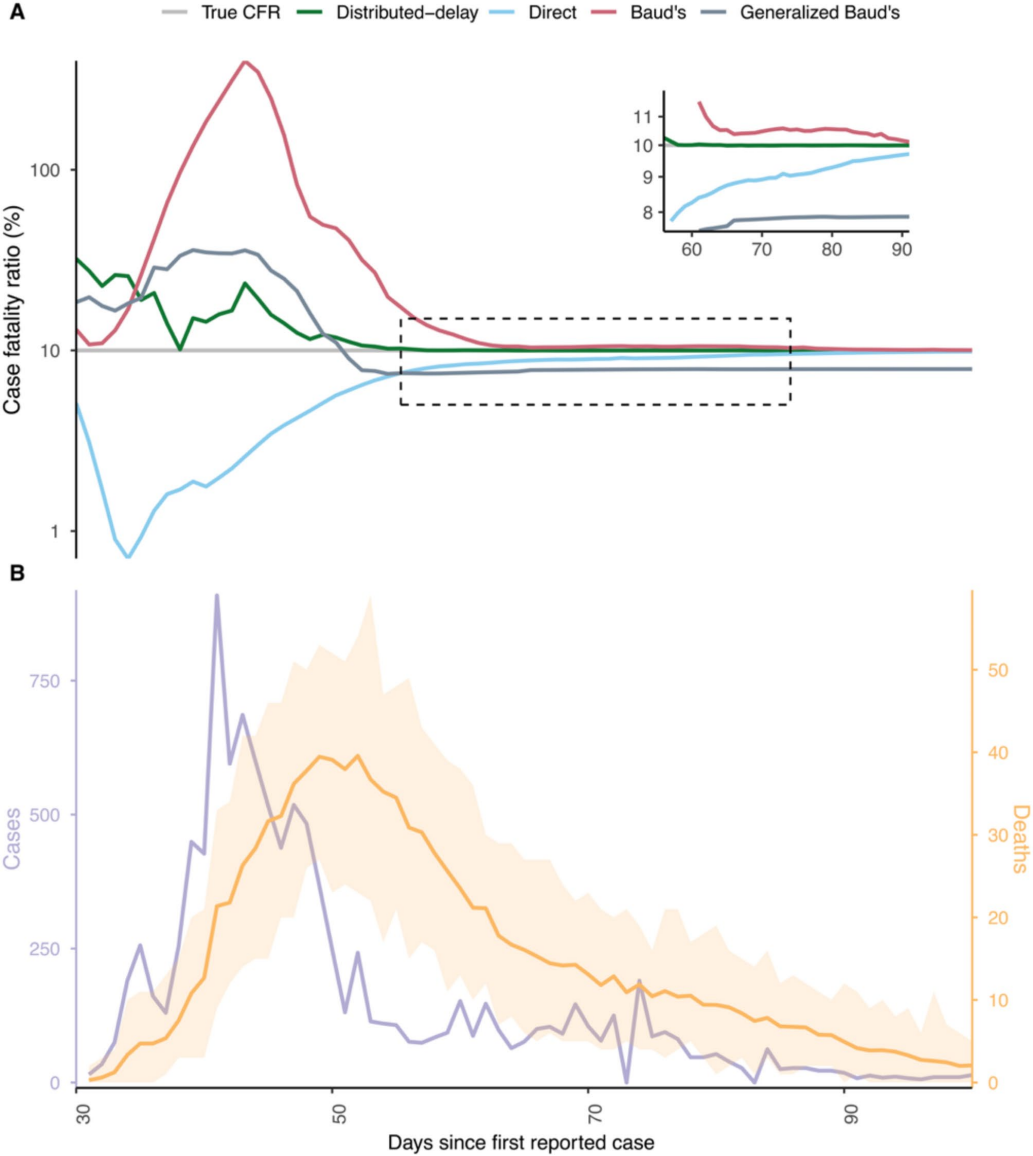
Comparison of CFR estimation methods on simulated data. Illustration of a distributed-delay method for simulated death data generated with arbitrarily chosen parameter values; case fatality ratio of $\:10\%$ case-to-death time parameters, a mean of $\:m=8.6$ days, and standard deviation of $\:s=6.7$ days. (**A**) Expected case fatality curve (green) obtained by fitting 100 realizations of simulated data using the distributed-delay method and the corresponding case fatality curve estimated by the direct method (blue), Baud’s method (red), and generalized Baud’s method (grey). The grey horizontal line represents the pre-defined case fatality ratio ($\:\lambda\:$) used to generate the death data. (**B**) Daily COVID-19 case-incidence (purple) in South Korea used to generate the simulated case fatalities (light orange – daily deaths) with the model. The inset figure shows the day our method converged to the true CFR.

We first present an instance where we test the distributed-delay method on simulation-generated case fatality data (see Methods). We assume that case-to-death times follow a lognormal distribution, as has been shown also to describe the variation of empirical case-to-death times^18^. Following Linton et al.^18^, and for illustration, we assume mean $\:m=8.6$ days, and standard deviation $\:s=6.7$ days of case-to-death times. For illustration, we assume the true CFR of 10% is also in the range of commonly observed CFR values, including COVID-19. We analyze and compare all the methods over the first 100 days from the onset of the epidemic (Fig. 2A). Figure 1A shows that the distributed-delay method estimates are more accurate than the other quantification methods except for the first few days owing to high stochasticity and data scarcity.

How long does it take for each method to converge to true CFR? For this, we consider a CFR estimate to have converged when the relative error stays less than 5% beyond the initial period of inherent stochasticity. Based on this, the distributed-delay method infers the assumed true CFR on day 56 of the outbreak (Fig. 2A, inset). Conversely, the direct method extremely underestimates the true CFR through the exponential phase and converges only on day 86 of the outbreak. Baud’s method greatly overestimates the true CFR through the exponential phase and converges only on day 66 (Fig. 2A).

We find that the Generalized Baud’s method performs better estimation than the direct and Baud’s method during the exponential phase, yet that it underestimates the CFR at the end of the outbreak—which is a phase in the outbreak where estimates by all the other methods become practically reliable. This is explained fundamentally by the lack of case-to-death times variation. The absence of variations in case-to-death times causes estimated case fatalities to be a shifted and scaled copy of cases. Since the actual case-to-death time distribution has a non-zero variance, the shape of actual case fatalities differs from that of cases. The estimated case fatality curve peaks sooner as it is right skewed. Therefore, fitting the estimated case fatalities to the actual case fatalities results in a lower mean case-to-death time as it reduces the residuals (see Discussion sections for more details).

How do the estimates of the distributed-delay method compare when the model parameters and distributions are varied? This naturally leads us to assess the robustness of the method.

### Robustness analysis on estimation accuracy

**Fig. 3:**
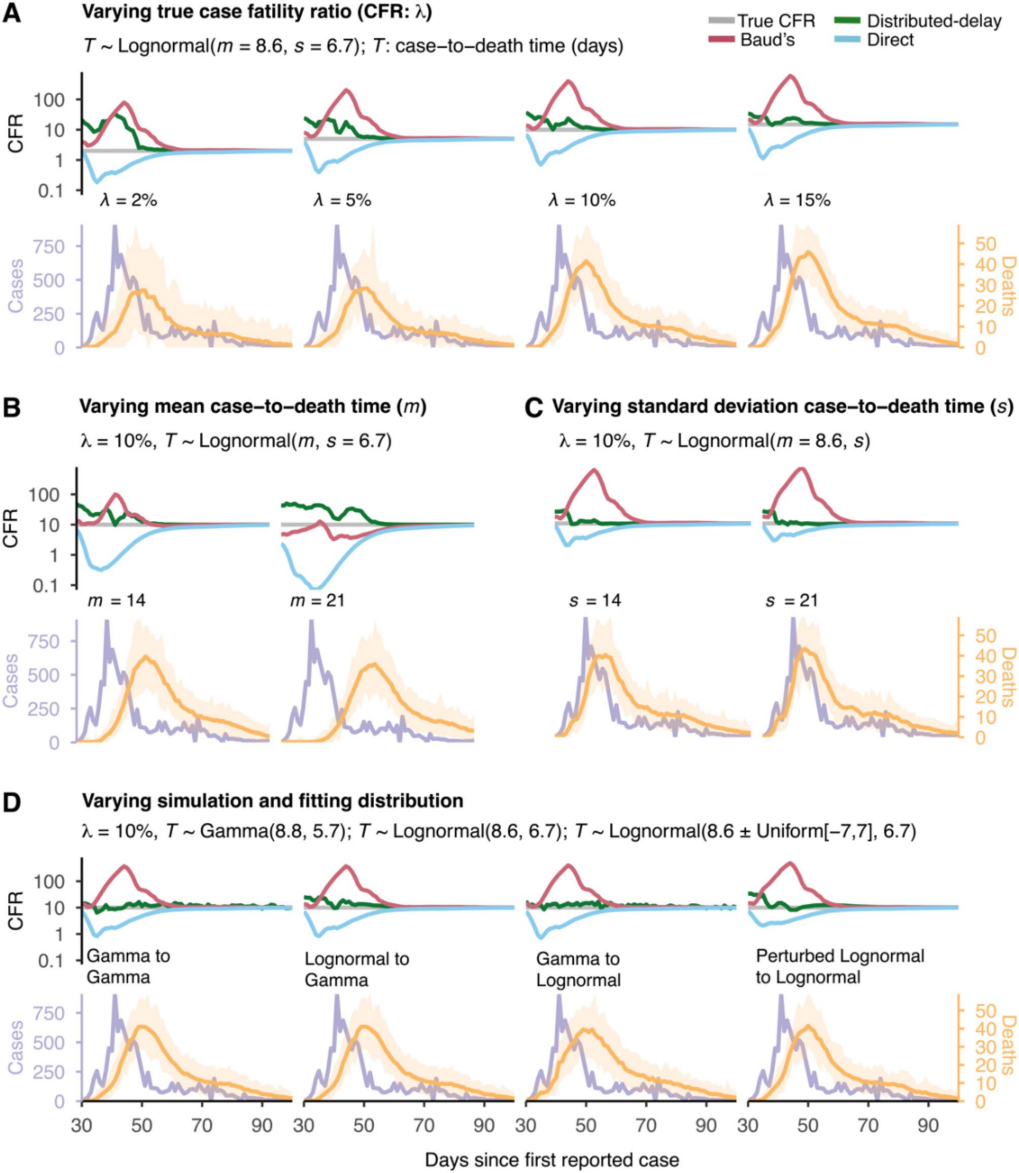
Robustness analysis under simulations scenarios: Distributed-delay method is robust. A comparison of the estimated case fatality ratio (CFR) (log scale) by distributed-delay method with other methods under various parameter settings and case-to-death times distributions. Each panel represents a simulation scenario in which we vary one of the model parameters, simulation, and (or) fitting distribution while holding others constant. (**A**) For $\:\lambda\:=\:\left(2,\:5,\:\text{10, 15}\right)\%$ and $\:m=\:8.6,\:s=\:6.7.$ (**B**) For $\:m=\:(14,\:21)$ and $\:s=\:6.7,\:\lambda\:=10\%.$ (**C**) For $\:s=\:(6.7,\:\text{14, 21})$ with$\:m=\:8.6,\:\lambda\:=10\%.$ (**D**) From left, Panel-1: Gamma to Gamma: data simulated and fitted with a gamma distribution; Panel-2: Gamma to lognormal: data simulated with gamma but fitted with a lognormal distribution; Panel-3: Lognormal to gamma: data simulated with lognormal but fitted with a gamma distribution. Panel-4: Perturbed lognormal to lognormal: data simulated with a perturbed lognormal distribution (i.e., $\:m\pm\:[-\text{7, 7}]$) but fitted with lognormal distribution gamma, the assumed parameters are $\:m=8.8$, $\:s=5.7$, $\:\lambda\:=10\%.$ For the lognormal, the assumed parameters are $\:m=\:8.6,\:s=\:6.7,\:\lambda\:=10\%$. We made 100 simulations and fitting runs at a daily scale for each case for the defined period.

Robustness analyses confirm that the distributed-delay method consistently provides better CFR estimates than the direct and Baud methods (Fig. 3).

We examine the performance of these methods by varying each of the parameters (see Table 2 for details), $\:\lambda\:$ (Fig. 3A), $\:m$ (Fig. 3B), and $\:s$ (Fig. 3C), and the simulation- and fitting distributions (Fig. 3D). The distributed-delay method provides more accurate estimates than other methods, irrespective of the parameter space.

The estimate of our method remains consistent even if the actual case-to-death times distribution is different from the one with which we fit the data (Fig. 3D; Lognormal to Gamma, Gamma to Lognormal, Perturbed Lognormal to Lognormal). This demonstrates that the method is robust and provides good estimates even if the true distribution does not closely match the assumed distribution. The case of perturbed lognormal (Fig. 3D, Perturbed Lognormal to Lognormal ) reflecting the reporting delays (or censoring bias) underpins that the method is insensitive to noise in the empirical data. For gamma-distributed case-to-death times (Fig. 3D; Gamma to Gamma), while our method’s estimates remain superior to others, its goodness-of-fit (R²) is lower compared to the lognormal distribution (Supplementary; Fig. S1).

To further evaluate the robustness and generalizability of the proposed method, we tested it on simulated fatality datasets derived from COVID-19 time-series case data from multiple countries, including Italy and Brazil. The results remained consistent with our initial findings, highlighting the reliability of the method across different datasets and scenarios (see Supplementary; Fig. S3).

### Robustness analysis on convergence time

**Fig. 4:**
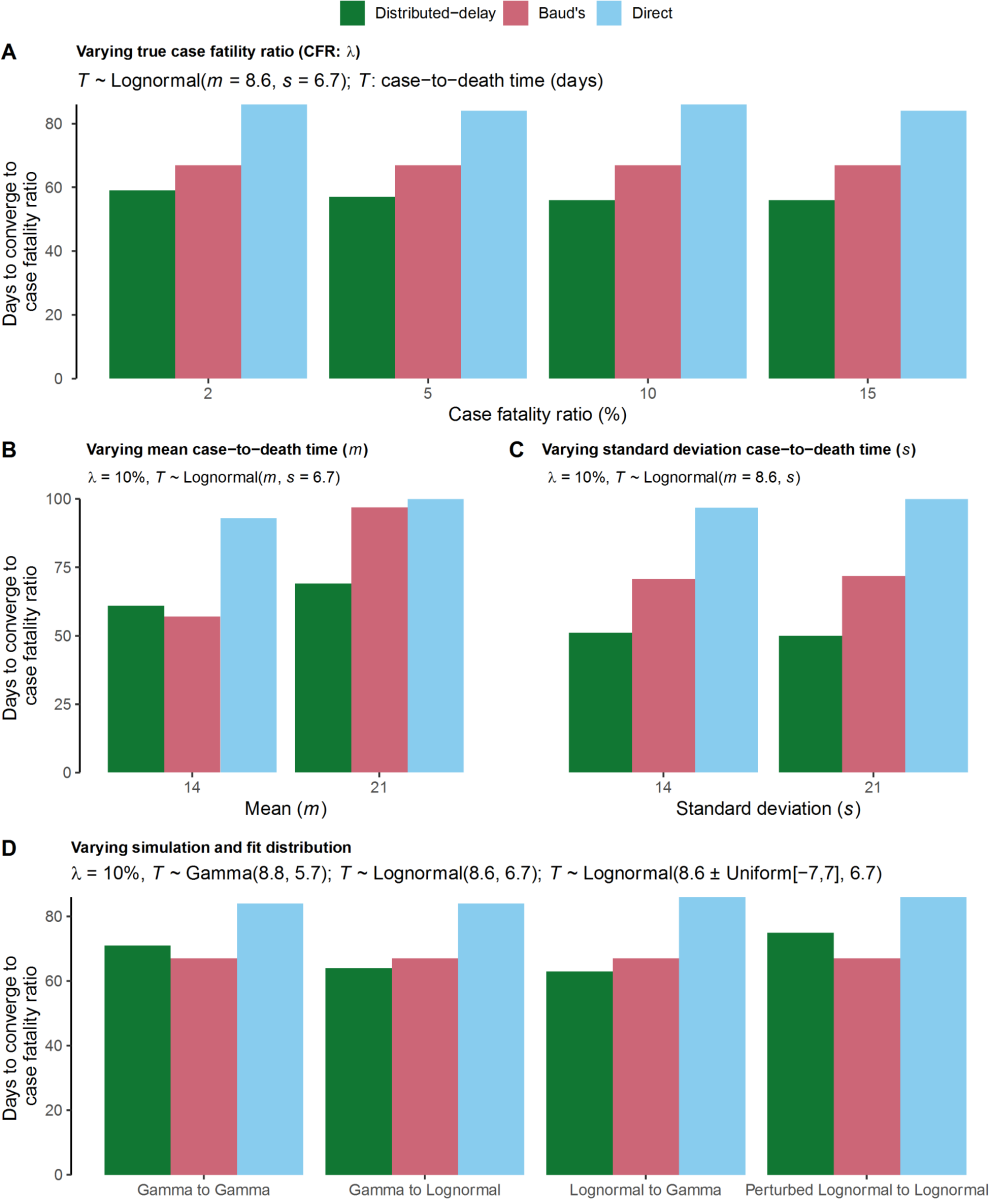
Robustness analysis on convergence time. Each panel represents a simulation scenario w.r.t constant and varying parameters and (or) distribution. The horizontal axis represents the assumed parameters and (or) distribution scenario. The vertical axis shows the number of days taken by a method to converge to the true CFR (%), considering a threshold relative error (RE) < 5%. (**A**) For$\:\lambda\:=\:(2,\:5,\:10,\:15)$and$\:m=\:8.6,\:\:s=\:6.7$ (**B**) For$\:m=\:(14,\:21)$and$\:s=\:6.7,\:\:\lambda\:=10\%$ (**C**) For$\:s=\:(6.7,\:14,\:21)$with$\:m=\:8.6,\:\lambda\:=10\%$ (**D**) From left, panel-1: Gamma to Gamma: data simulated and fitted with a gamma distribution; Panel-2: Gamma to lognormal: data simulated with gamma but fitted with a lognormal distribution; Panel-3: Lognormal to gamma: data simulated with lognormal but fitted with a gamma distribution. Panel-4: Perturbed lognormal to lognormal: data simulated with a perturbed lognormal distribution (i.e., $\:m\pm\:[-\text{7, 7}]$) but fitted with lognormal distribution gamma, the assumed parameters are$\:m=8.8$, $\:s=5.7$, $\:\lambda\:=10\%$. For the lognormal, the assumed parameters are$\:m=\:8.6,\:\:s=\:6.7,\:\:\lambda\:=10\%$. In each case, we made$\:100$ simulations and fitting run on a daily scale for the defined period.

We estimate the time (days) each method takes to converge to the true CFR for each simulation scenario listed in Table 2. For that, we again define convergence to be met at a point when the relative error of the estimates remains less than 5%. Depending on the simulation scenario, the distributed-delay method converges 7 to 45 days earlier than the direct method (Fig. 4). Also, the method’s convergence time remains superior to Baud’s methods except for three instances (Fig. 4B; $\:m=14\:$, Fig. 4D; Gamma to Gamma, Fig. 4D; Perturbed Lognormal to Lognormal). These analyses highlight the robustness of our approach concerning the convergence time.

These findings confirm the superiority of our approach in controlled settings. However, examining how well the method performs on empirical data is crucial, which we explore in the following sub-section.

### Application to empirical COVID-19 data

We analyze COVID-19 data from 34 countries (see Supplementary; Table S1). Unlike simulations, real-world case fatalities are typically unknown before the outbreak wanes or when complete information on the outcomes of cases is available. Typically, the eventual (or true) CFR is averaged across the whole duration of an outbreak and may vary over time as reported for COVID-19. We restrict our analysis to the assumption that CFR remains constant throughout the epidemic (see Discussions). We thus choose the CFR estimates of each method at two time points; (i) early-phase estimations: We define the estimates of day 30 (since the first reported death) as early-phase CFR, and (ii) Eventual CFR: the estimates on day 100 of the pandemic.

**Fig. 5:**
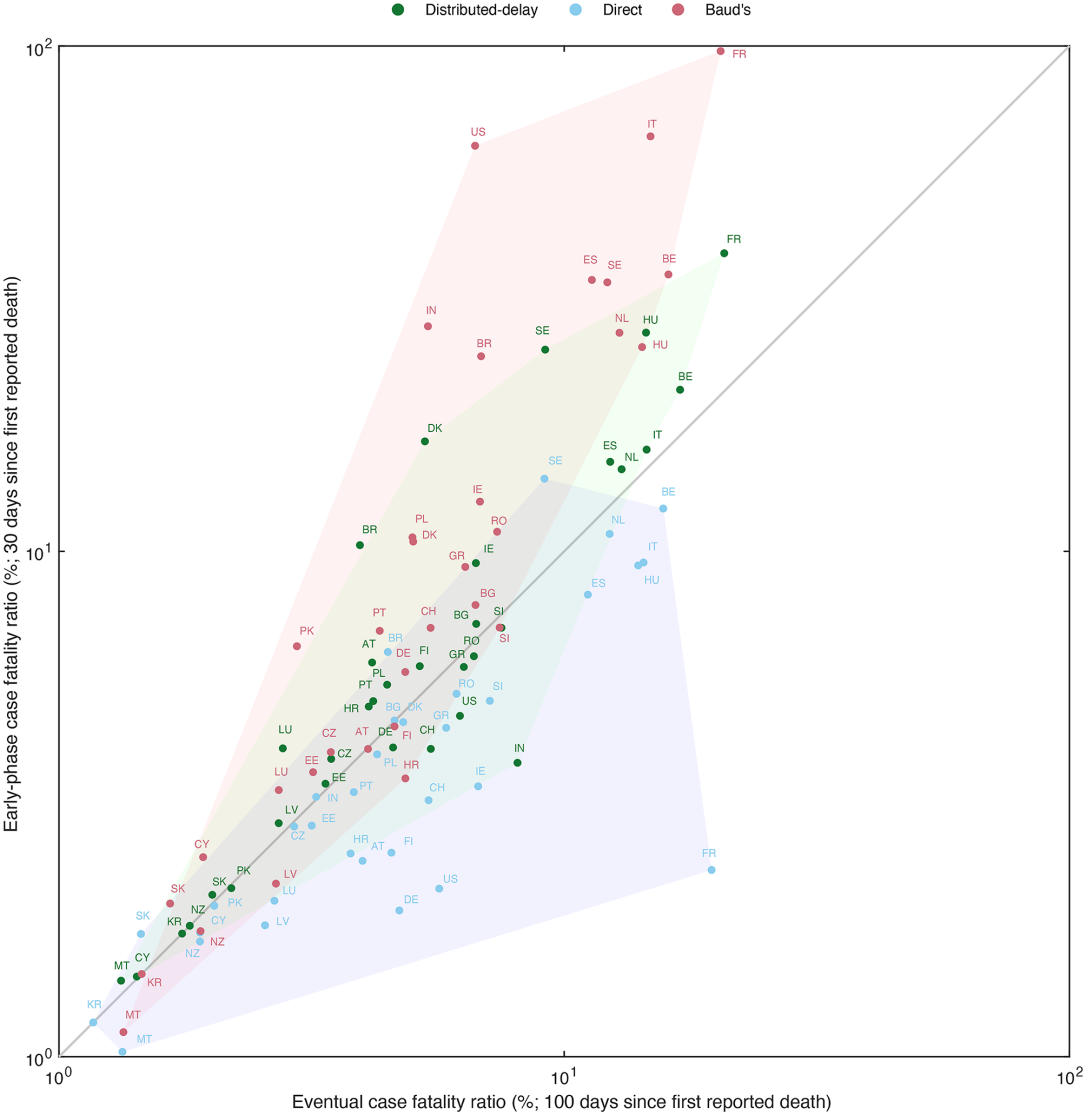
Comparison of CFR estimation methods on empirical COVID-19 data. Comparison of eventual (horizontal-axis) and the early-phase case fatality ratio (vertical-axis) with the distributed-delay method (green), the direct method (blue), and Baud’s method (red) on a log scale for the 34 countries. Each country is represented by its two-letter International Organization for Standardization (ISO) code. The green convex hull of the distributed-delay method shows that the early-phase case fatality is higher than the eventual CFR. The blue convex hull of the direct method’s early-phase CFR largely lies below the diagonal line, showing a substantial underestimation of empirical CFR. Similar to simulations, early-phase CFR estimated from Baud’s (red convex hull) method predominantly lies above the diagonal, depicting more significant errors than the other methods.

To compare the estimates from respective methods, we assess their symmetry along a diagonal line by considering that the method aligning closely to the diagonal line converges sooner to the eventual CFR in the considered time window. We observe that the estimates of the distributed-delay method (green convex hull) closely align with the diagonal and remain between those of the direct (blue convex hull) and Baud’s method (red convex hull) (Fig. 5). The early-phase estimations by the distributed-delay method consistently approach the eventual CFR more than others. Again, for illustration and analogous to simulation-based temporal CFR curves, the country-wise (of European Union countries) empirical CFR curves of each method are provided in Figure S6 of Supplementary material.

### Relationship between empirical case fatality ratios and case-to-death times

**Fig. 6:**
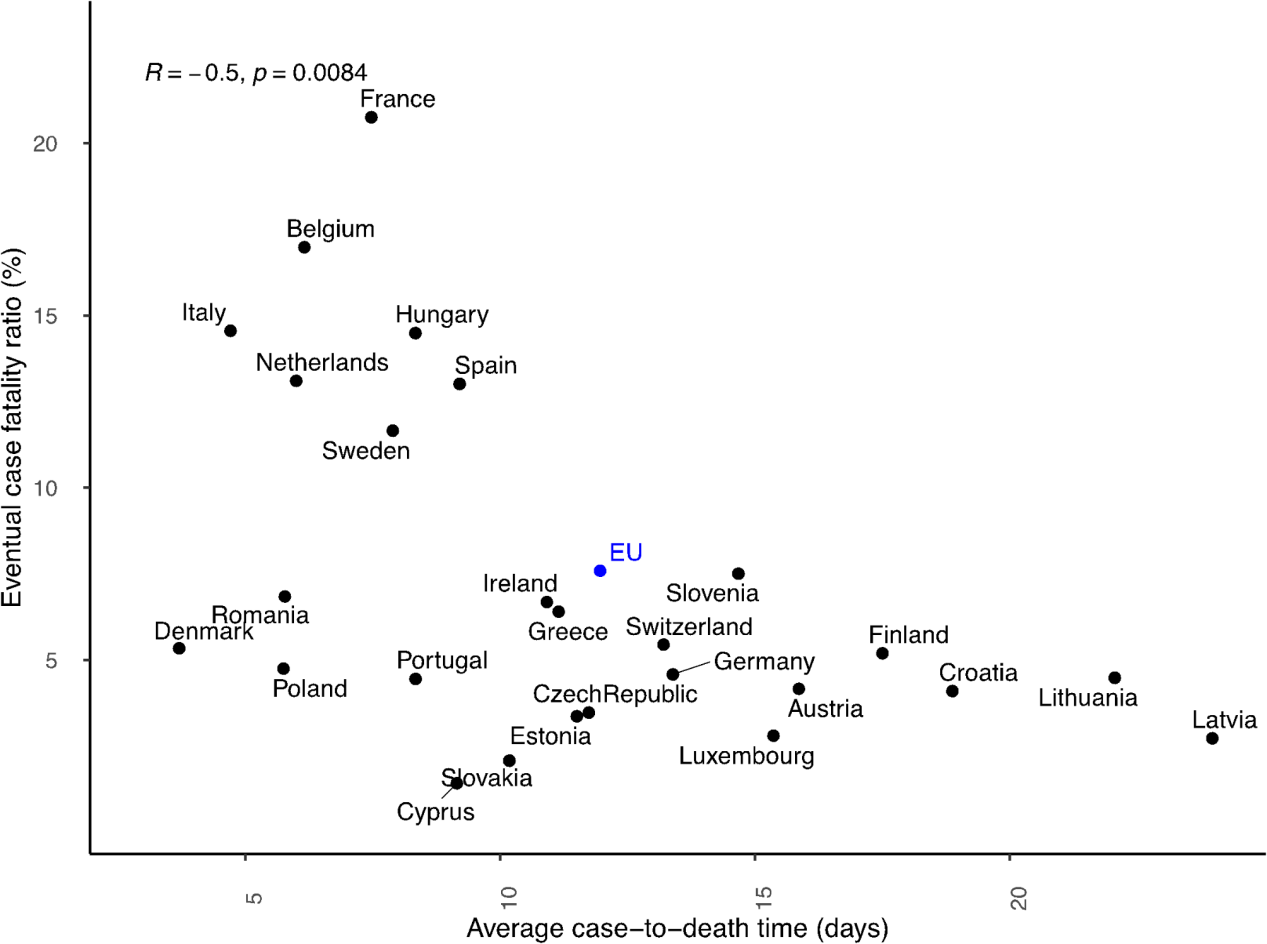
Association between the expected case-to-death times and the eventual case-fatality ratio. The horizontal axis represents the mean case-to-death time (days) and the corresponding eventual case-fatality ratio (%) of the European Union (EU) and each of its member countries. The eventual CFR corresponds to the estimates of day 100 of the pandemic in each country.

In addition to the CFR, we also estimate, from our method, the expected case-to-death time for the member countries of the European Union (EU) (Fig. 6). We further examine the association between eventual CFR and case-to-death time for the EU and each country. A significantly $\:(p\sim0.0084)$ negative correlation$\:(R\:\sim-0.5)$ between the average case-to-death time and the eventual case-fatality ratio was found. This association reveals that countries with more severe outbreaks $\:(CFR >10\%)$ generally have had shorter case-to-death time $\:(<7\:days)$ for COVID-19 (Fig. 6). These are more tangible for countries such as France, Belgium, Italy, Netherlands, Spain, Sweden, etc.

## Discussion

The case fatality ratio (CFR) is a crucial epidemiological measure used to gauge disease severity during new pandemics or epidemics and to evaluate the necessity for control and intervention strategies. At a later stage of a pandemic, complementary estimates of the infection fatality ratio (IFR) become available, which take into account all infections, not just clinical cases. This research shows how well-established and routinely used crude CFR estimators (direct method and Baud’s method) can be associated with notable biases due to their inherently simplified assumptions.

In this study, we have presented a new CFR quantification method that provides improved estimates during disease outbreaks by demonstrating how our approach addresses the key limitations of the above methods and improves CFR estimation using publicly available data. We have also shown the importance of accounting for variable case-to-death times and the distribution of their relative frequencies and that distribution parameters (e.g., mean and standard deviation) along with CFR can be inferred by fitting modeled case fatalities to actual case fatalities. We showed that with these relaxed and more realistic assumptions, estimation errors and convergence times can be improved compared to the direct and Baud’s methods that make oversimplified assumptions on case-to-death times.

The method achieves reliable CFR estimates typically after 40–80 days from the onset of the outbreak, depending on the outbreak size and the case-to-death parameters of the distribution. As simulations demonstrate, it converges up to 45 days earlier than the other methods. The method not only accommodates case-to-death times parametric distribution but also the variability and the distortions in these distributions, as demonstrated in our robustness analyses (Fig. 3).

Beyond the improved CFR estimation, the method provides interesting insights into the relationship between CFR and the expected case-to-death time. One interesting observation is a significantly negative correlation between the estimated CFR and case-to-death times in empirical COVID-19 data (Fig. 6). Is this a characteristic of COVID-19 specifically, or does it reflect a general principle across multiple diseases? Understanding the generality of this relationship can be helpful for rapid CFR assessment early on in outbreaks.

The distributed-delay method builds on four key components. (i) The case-to-death time distribution. This is essential, as was demonstrated through simulations and for empirical COVID-19 data across multiple countries. (ii) A flexibility in accommodating any case-to-death time distribution (as it could potentially differ between outbreaks). (iii) Quantification relies solely on information from the outbreak under consideration. (iv) It requires time series data on reported cases and fatalities that typically are publicly available. These components also contribute to an improved applicability relative to other CFR quantification methods (Table 1).

Quantifying CFR and case-to-death times parameters through the distributed-delay method requires fitting modeled case fatalities into data. Importantly, we fitted the modeled daily case fatalities to daily reported case fatalities data here. While it is easier for any minimization algorithm to settle on residuals minima with respect to cumulative case fatality data – as this involves aligning two sigmoid-shaped curves (i.e., actual and modeled cumulative case fatalities)—it is also prone to settle on false minima and therefore introducing misleading errors. The true minima with respect to daily case fatality data are better defined and, thus, the better option out of the two. Although minimizing with respect to cumulative case fatalities data may, at first sight, appear statistically more appealing (e.g., as it typically generates higher R²), it may fall short in capturing the true underlying relationships of CFR, leading to false CFR and distribution parameter estimations.

The literature offers a range of parametric and non-parametric quantification methods attempting to correct CFR estimations. Indeed, many of these studies incorporate variations in case-to-death times^13,15,17,26–28^. In these studies, however, the shape of case-to-death time distributions depends on parameter prerequisites inferred from other known outbreaks, which may not necessarily be the same for a novel disease. Even if the distribution parameters are available from the disease under consideration, the predicted CFR tends to be sensitive to the choice of distribution parameters and may not accurately reflect the underlying population heterogeneities. Additionally, a few of these methods require information that is not available during the outbreaks for which they are intended and are therefore not applicable when CFR information is needed the most^5,29^. For instance, the modified Kaplan–Meier method described by Ghani et al.^5^. , relies on individual case data, including the dates of hospitalization and death or recovery, to estimate the CFR. In contrast, the distributed-delay method requires only publicly reported data on cases and deaths and no prior information on case-to-death times distribution.

One of the central aspects of the distributed-delay method is that it flexibly considers the distribution of case-to-death times. Only a few earlier studies have acknowledged the importance of this aspect in quantifying CFR^17,28,30^. One of these studies, by Nishiura et al.^17^, presents a method that may provide earlier estimates of CFR than the one presented here – under optimal conditions. However, the quantification method by Nishiura et al. often falls short because it requires inference on case-to-death time parameter values from past outbreaks. Considering the many unknowns associated with novel outbreaks, such a method is rarely applicable in new epidemiological situations, which are also situations where information on outbreak severity is often needed the most.

A notable strength of this study lies in its novel methodology, which, to our knowledge, is the first to simultaneously estimate the case-to-death times (or delay) distribution parameters and case fatality ratio (CFR) directly from aggregate data. This approach circumvents the reliance on external delay distribution estimates, which may not adequately capture outbreak-specific factors such as demographic variations, healthcare infrastructure, or reporting practices. These outbreak-specific factors are critical for accurate estimation, particularly when individual-level data or precise case-to-death delay distributions are unavailable.

While recognizing the strengths of our method, identifying the case-to-death (or delay) distribution from aggregate data can be challenging in the early epidemic stages, especially when cases are growing exponentially. For instance, the collinearity between the distribution parameters and CFR may limit precision in these phases. Nevertheless, our simulation results indicate that the proposed method performs robustly even under these conditions, providing reasonable estimates. Furthermore, as additional data are collected over time, the estimation uncertainty diminishes substantially, even during periods of exponential growth (Supplementary; Fig. S3B).

It is worth noting that the uncertainty in the early phase CFR estimates may be large (Supplementary; Fig S3). This is because during the early phase of an outbreak, the number of reported cases is often small, and the corresponding number of fatalities is subject to considerable randomness. This randomness stems from two factors: the probabilistic nature of death resulting from infection and the variability in the timing of fatalities due to the distribution of case-to-death times. Formally, the variability in the number of deaths arising from $\:n$ cases can be expressed as $\:\frac{1}{\sqrt{n.p}}\sqrt{1-p}$, where $\:p$ is the probability of fatality. When $\:p$ is small, as it often is for novel diseases, the uncertainty increases further with the number of deaths given $\:\frac{1}{\sqrt{n.p}}$, and a high number of cases is required to offset this noise. The additional uncertainty introduced by the timing of deaths further amplifies this variability in the early phase of outbreaks. As the outbreak progresses and case numbers increase, the uncertainty decreases, as reflected in the narrowing of the uncertainty band over time (Figure S3).

This study also highlights an important trade-off: relying on external estimates may introduce biases due to mismatch with outbreak-specific dynamics while deriving estimates directly from aggregate data ensures specificity but may come with higher uncertainty in early stages, as noted above. While our work does not directly evaluate the comparative advantages of these approaches, it provides a foundation for future studies to explore this trade-off under diverse outbreak scenarios. Investigating the conditions under which outbreak-specific estimates become preferable to external estimates—through simulations or empirical comparisons—would represent an important direction for advancing this field.

The distributed-delay method introduces a higher level of complexity than the oversimplified direct or Baud’s methods, and these oversimplified quantification methods typically do not involve any minimization problem. An expected consequence of this increase in complexity and optimization requirement is that more extensive computational resources are required for CFR quantification. The realized computational time of the distributed-delay method is variable. It typically depends on multiple factors, including case incidence, case fatalities incidence, and the degree of required tuning of the parameters of the optimization algorithm. (see Supplementary: Computational cost of distributed-delay method). Although a steep trade-off may be associated with computational cost, this consideration must not undermine the necessity of accurate quantifications of CFR.

As noted in the results (Fig. 2), the Generalized Baud’s method underestimates the parameters $\:\lambda\:$ and $\:m$. We find that this is because the shape of the death curve is fixed since $\:s\to\:0$ and does not follow the shape of a death curve generated from a case-to-death time distribution. When the case-to-death time distribution has a long tail, it generates many small case-to-death times and a few potentially very long ones. As a result, the realized fatalities data is less sharply peaked than the case data, and the time between the cases and fatalities is often smaller than the average case-to-death time. When we assume a case-to-death time distribution with zero variance (i.e., a delta distribution) as in the Generalized Baud’s method, the estimated fatalities become a time-shifted copy of the curve of the case, which has a fundamentally different shape than the actual fatality curve (see Supplementary; Fig. S5). As a result, the peak of the Generalized Baud’s estimations, given the assumed, $\:m$ occurs later than the peak of the data, and minimums are therefore found by underestimating the average case-to-death time when the two peaks align. The same principle applies to $\:\lambda,$ but in terms of the areas under the curve, as this equals the cumulative number of deaths. Residuals are not minimized for the true $\:\lambda\:$, as this narrower shape requires many fatalities around the peak.

Any estimator should ideally be unbiased, meaning that variation around the average estimate is symmetrical and that the average estimate is correct in the limit of infinite samples. We have observed a slight overestimation in CFR estimates in the very early phase. From additional analyses, we conclude, however, that the estimator is unbiased in itself but that the average of realizations is systematically repelled from the CFR boundaries as the distribution of realizations becomes, as expected, skewed near the boundaries of the CFR range, i.e., [0, 1] (Supplementary; Fig. S4). Such effects may also arise depending on the choice of minimization algorithm. Applying least squares in sparse data settings, as we did in this study, could generate these types of boundary effects. A more flexible method, like maximum likelihood, could minimize such boundary effects, which we leave for future work.

This study provides point estimates of the estimated parameters. Uncertainty in the estimated CFR may arise from data variability and model assumptions, particularly in early outbreak phases when case-to-death dynamics are not fully observed. Future work can incorporate techniques such as bootstrapping to derive uncertainty bounds such as confidence intervals when using our distributed-delay method.

Our study assumes that the CFR is constant throughout an epidemic, providing a controlled framework to evaluate the convergence behavior of estimation methods. The observed variation in the estimated CFR over time, as shown in Figs. 1 and 2, reflects the error inherent in the estimation process rather than any inconsistency in the true underlying CFR, which is held constant in the simulations. This variation highlights the methods’ ability to converge toward the true CFR, particularly during the exponential growth period of the outbreak.

The CFR, however, can vary over time for various reasons. For example, early in an outbreak of a novel pathogen, severe infections are disproportionately likely to be accounted for as a case due to limited testing or lower disease familiarity among patients and the health system^31,32^. The lack of knowledge about novel infectious agents can also result in many case fatalities initially. Factors like healthcare system readiness, demography, local disease dynamics, testing, tracking systems, and data reporting can influence ongoing disease severity estimates^32^. This is also dependent on how one defines CFR. The conventional concept of the CFR for a given disease outbreak, typically obtained in hindsight past the outbreak, refers to the long-term average CFR. One implication of assuming a constant, or conventional, CFR when comparing the performance of the respective quantification methods on empirical data is that no point of reference exists except for the long-term average. The performance on empirical data was adjudged here concerning how well the long-term average CFR can be inferred by the respective methods already early in the outbreak (Fig. 5).

Capturing the real-time (or time-dependent) CFR requires information on case fatality or recovery for each individual reported as a disease case and the distribution of associated case-to-death times. Such detailed information, particularly during the early phases of novel outbreaks, is often unavailable. Timely reporting of such individualized case data on case fatality or recovery is crucial for accurate disease severity assessment throughout an outbreak. We emphasize that reporting of individualized data should be recommended as it would help enable further improved disease severity quantification early in outbreaks.

While the method indirectly accounts for demographic heterogeneity through aggregated cases and deaths data, it does not explicitly estimate CFR contributions from specific subgroups (e.g., age or gender). Extending the method to stratified analyses, where cases and fatalities are grouped by key demographic variables, could enable more precise subgroup-specific estimates. By applying the distributed-delay method to each subgroup and aggregating the results, a weighted average CFR for the whole population could be obtained, accounting for demographic differences. Such extensions would allow for evaluating the specific contributions of demographic heterogeneities to overall CFR estimates, providing valuable insights for targeted public health interventions.

Recognizing the earlier considerations, the distributed-delay method offers room for further improvements. Future research should focus on modeling the case fatality ratio as a time-varying quantity, further incorporating factors like population demographics (age and gender), and assessing the influence of interventions. Our analysis can be further extended by exploring a broader range of case-to-death time distributions, such as the Weibull distribution, conducting systematic comparisons with similar methodological approaches (e.g., Nishiura et al.^17^), and applying the proposed framework to historical epidemic datasets. These extensions would provide valuable insights into the generalizability of the method across diverse outbreak scenarios and further evaluate its practical utility for real-world applications.

As noted by Lipsitch et al. (2015)^20^, CFR estimates are influenced by multiple biases, including delays in reporting deaths, underascertainment of mild cases, changes in testing practices, and demographic composition of cases. While our study focuses primarily on addressing the delay bias using aggregated time-series data on confirmed cases and death data in improving CFR compared to the widely used direct method in an ongoing outbreak, future work should aim to integrate these dynamic factors to provide a more comprehensive framework for CFR estimation.

In this analysis, we did not factor in the effects of new variants of a pathogen because it mainly addresses the early estimation of overall CFR in an outbreak—when, most likely, only one variant would circulate. If data were stratified by variant (e.g., through genomic surveillance), our method could be applied separately to assess the variant-specific CFR. This would provide valuable insights into how different variants influence disease severity over time.

In conclusion, the distributed-delay method yields more accurate CFR estimates than earlier alternative methods. It adeptly accommodates realistic variability in case-to-death times, revealing limitations in previous methods. Operating solely on data from the specific epidemic under consideration, the method avoids reliance on parametric assumptions, making it useful for, for instance, outbreaks of novel pathogens. The new method could prove valuable in future outbreaks and for pandemic preparedness, if further developed, to estimate real-time CFR effectively.

## Electronic supplementary material

Below is the link to the electronic supplementary material.

Supplementary Material 1

## Data Availability

The data used in the analysis is open source and referenced. The output data generated during the current study are available from the corresponding author on reasonable request.

## References

[1] Hashmi, H. A. S. & Asif, H. M. Early detection and assessment of covid-19. *Front. Med.* **7**, (2020). 10.3389/fmed.2020.00311 PMC7296153 32582748

[2] Steele, L., Orefuwa, E. & Dickmann, P. Drivers of earlier infectious disease outbreak detection: a systematic literature review. *Int. J. Infect. Dis.* **53**, 15–20 (2016). 27777092 10.1016/j.ijid.2016.10.005

[3] Goyal, D. K., Mansab, F., Iqbal, A. & Bhatti, S. Early intervention likely improves mortality in COVID-19 infection. *Clin. Med. Lond.*10.7861/clinmed.2020-0214 (2020). 32357975 10.7861/clinmed.2020-0214 PMC7354047

[4] Giesecke, J. *Modern Infectious Disease Epidemiology (3rd Ed.)*. (2017).

[5] Ghani, A. C. et al. Methods for estimating the case fatality ratio for a novel, emerging infectious disease. *Am. J. Epidemiol.* **162**, 479–486 (2005). 16076827 10.1093/aje/kwi230 PMC7109816

[6] Kucharski, A. J. & Edmunds, W. J. Case fatality rate for Ebola virus disease in West Africa. *Lancet* **384**, 1260 (2014). 25260235 10.1016/S0140-6736(14)61706-2

[7] Russell, T. W. et al. Estimating the infection and case fatality ratio for coronavirus disease (COVID-19) using age-adjusted data from the outbreak on the Diamond Princess cruise ship, February 2020. *Eurosurveillance* **25**, 2000256 (2020). 32234121 10.2807/1560-7917.ES.2020.25.12.2000256 PMC7118348

[8] Lipsitch, M. Estimating case fatality rates of COVID-19. *Lancet Infect. Dis.* **20**, 775 (2020). 32243813 10.1016/S1473-3099(20)30245-0 PMC7270796

[9] Baud, D. et al. Real estimates of mortality following COVID-19 infection. *Lancet Infect. Dis.* **20**, 773 (2020). 32171390 10.1016/S1473-3099(20)30195-X PMC7118515

[10] Lui, G. C. Y. et al. Significantly lower case-fatality ratio of coronavirus disease 2019 (COVID-19) than severe acute respiratory syndrome (SARS) in Hong Kong—a territory-wide cohort study. *Clin. Infect. Dis. Off Publ Infect. Dis. Soc. Am.*. 10.1093/cid/ciaa1187 PMC7543259 33005933

[11] Hu, W. et al. Estimation of COVID-19 case fatality ratio based on a bi-directional correction method. *medRxiv* (2020).

[12] Shim, E., Mizumoto, K., Choi, W. & Chowell, G. Estimating the risk of COVID-19 death during the course of the outbreak in Korea, February–May 2020. *J. Clin. Med.* **9**, 1641 (2020). 32485871 10.3390/jcm9061641 PMC7356403

[13] Wilson, N., Kvalsvig, A., Barnard, L. T. & Baker, M. G. Case-fatality risk estimates for COVID-19 calculated by using a lag time for fatality. *Emerg. Infect. Dis.* **26**, 1339 (2020). 32168463 10.3201/eid2606.200320 PMC7258483

[14] Angelopoulos, A. N., Pathak, R., Varma, R. & Jordan, M. I. On identifying and mitigating bias in the estimation of the COVID-19 case fatality rate. *Spec. Issue 1-COVID-19* (2020).

[15] Thomas, B. S. & Marks, N. A. Estimating the case fatality ratio for COVID-19 using a time-shifted distribution analysis. *medRxiv* (2020). 10.1017/S0950268821001709 34275506

[16] Spychalski, P., Błażyńska-Spychalska, A. & Kobiela, J. Estimating case fatality rates of COVID-19. *Lancet Infect. Dis.* (2020). 10.1016/S1473-3099(20)30246-2 PMC7270730 32243815

[17] Nishiura, H., Klinkenberg, D., Roberts, M. & Heesterbeek, J. A. Early epidemiological assessment of the virulence of emerging infectious diseases: a case study of an influenza pandemic. *PloS One*. **4**, e6852 (2009). 19718434 10.1371/journal.pone.0006852 PMC2729920

[18] Linton, N. M. et al. Incubation period and other epidemiological characteristics of 2019 novel coronavirus infections with right truncation: a statistical analysis of publicly available case data. *J. Clin. Med.* **9**, 538 (2020). 32079150 10.3390/jcm9020538 PMC7074197

[19] Jewell, N. P. et al. Non-parametric estimation of the case fatality ratio with competing risks data: an application to severe Acute Respiratory Syndrome (SARS). *Stat. Med.* **26**, 1982–1998 (2007). 16981181 10.1002/sim.2691 PMC7169492

[20] Lipsitch, M. et al. Potential biases in estimating absolute and relative case-fatality risks during outbreaks. *PLoS Negl. Trop. Dis.* **9**, e0003846 (2015). 26181387 10.1371/journal.pntd.0003846 PMC4504518

[21] Kennedy, J. & Eberhart, R. *Particle Swarm Optimization* vol. 4, 1942–1948 (IEEE, 1995).

[22] Ritchie, H., Ortiz-Ospina, E. & Beltekian, D. Our world in data-coronavirus (COVID-19) testing. *Httpsourworldindata Orgcoronavirus-Test Last Accessed Oct.* **30**, 2020 (2020).

[23] MATLAB. *9.8.0.1359463 (R2020a) Update 1*. (The MathWorks Inc., 2020).

[24] Nelder, J. A. & Mead, R. A simplex method for function minimization. *Comput. J.* **7**, 308–313 (1965).

[25] Particle Swarm Optimization. In *Computational Intelligence* 289–358 10.1002/9780470512517.ch16 (Wiley, 2007).

[26] Russell, T. W. et al. Reconstructing the early global dynamics of under-ascertained COVID-19 cases and infections. *BMC Med.* **18**, 332 (2020). 33087179 10.1186/s12916-020-01790-9 PMC7577796

[27] Undurraga, E. A., Chowell, G. & Mizumoto, K. COVID-19 case fatality risk by age and gender in a high testing setting in Latin America: Chile, March–August 2020. *Infect. Dis. Poverty*. **10**, 1–11 (2021). 33531085 10.1186/s40249-020-00785-1 PMC7854021

[28] Garske, T. et al. Assessing the severity of the novel influenza A/H1N1 pandemic. *BMJ* **339** (2009). 10.1136/bmj.b2840 19602714

[29] Wilson, N. & Baker, M. G. The emerging influenza pandemic: estimating the case fatality ratio. *Eurosurveillance* **14**, 19255 (2009). 19573509

[30] WHO. Estimating Mortality from COVID-19: Scientific Brief, 4 August 2020. 1. (2020).

[31] Dudel, C. et al. Monitoring trends and differences in COVID-19 case-fatality rates using decomposition methods: contributions of age structure and age-specific fatality. *PloS One* **15**, (2020). 10.1371/journal.pone.0238904 PMC7482960 32913365

[32] Perez-Saez, J. et al. Serology-informed estimates of SARS-CoV-2 infection fatality risk in Geneva, Switzerland. *Lancet Infect. Dis.* **21**, e69–e70 (2021). 32679085 10.1016/S1473-3099(20)30584-3 PMC7833057

