# Supplementary material for: Improving case fatality ratio estimates in ongoing pandemics through case-to-death time distribution analysis
Source: Sci Rep. 2025 Feb 13;15:5402. doi: 10.1038/s41598-025-89441-y (PMC11825655; doi:10.1038/s41598-025-89441-y)
Supplement: Supplementary file 1 — Supplementary Material 1 [file 41598_2025_89441_MOESM1_ESM.docx]

Supplementary Information

Improving case fatality ratio estimates in ongoing pandemics through case-to-death time distribution analysis

Zia Farooq*, ^a^ Henrik Sjödin, ^a^ Joacim Rocklöv, ^a,b^ Åke Brännström ^c,d^

1. Department of Epidemiology and Global Health, Umeå University, Umeå, 901 87, Sweden
2. Heidelberg institute of global health and Interdisciplinary centre for scientific computing, University of Heidelberg, Im Neuenheimer Feld 205, Heidelberg 69120, Germany
3. Department of Mathematics and Mathematical Statistics, Umeå University, Umeå, 901 87, Sweden
4. Complexity Science and Evolution Unit, Okinawa Institute of Science and Technology

Graduate University, Kunigami, Japan

**Corresponding author**

Zia Farooq, PhD

Postdoctoral Researcher,

target point F52, Floor 5,

Department of Epidemiology and Global Health,

Umeå University,

Umeå 901 87,

Sweden

Phone: +46735983174

Email: [zia.farooq@umu.se](mailto:zia.farooq@umu.se)

# Additional analysis and figures

**Figure S 1:** **R^2^ estimates for simulation scenarios.** *The curves of R^2^ under each simulation scenario are presented in the main text.*

**Figure S 2:** **Estimated mean (m) and Standard deviation (s) of case-to-death times distribution for simulation scenarios.** *The curve of estimated mean (m) and standard-deviation (s) of case-to-death times parameters of the distributed-delay method. The dashed horizontal lines represent the assumed parameters values used to simulate the data.*

**Figure S 3:** **Additional simulations from Italy and Brazil data.** *Illustration of a distributed-delay method for Italy (A and C) and Brazil (B and D) for simulated death data generated with arbitrarily chosen parameter values; case fatality ratio of* $10\%$ *case-to-death time parameters, a mean of* $m=8.6$ *days, and standard deviation of* $s=6.7$ *days. A & B) Expected case fatality curve (green) obtained by fitting 100 realizations of simulated data using the distributed-delay method and the corresponding case fatality curve estimated by the direct method (blue), Baud's method (red). The grey horizontal line represents the pre-defined case fatality ratio (*$\lambda$*) used to generate the death data. C & D) Daily COVID-19 case-incidence (purple) in Italy (C) and Brazil (D) used to generate the simulated case fatalities (light orange – daily deaths) with the model.*

**Figure S 4: Extreme case fatality ratio simulation scenarios.** *A) Simulated fatalities (average) for three instances of the case fatality ratios* $(0.1, 0.5, 0.9)$*. The dashed lines show the range(minimum-maximum) of simulated fatalities. B) The estimated case fatality ratios by the distributed-delay method for each scenario. The dashed lines represent the true CFR value used to simulate the fatalities.*


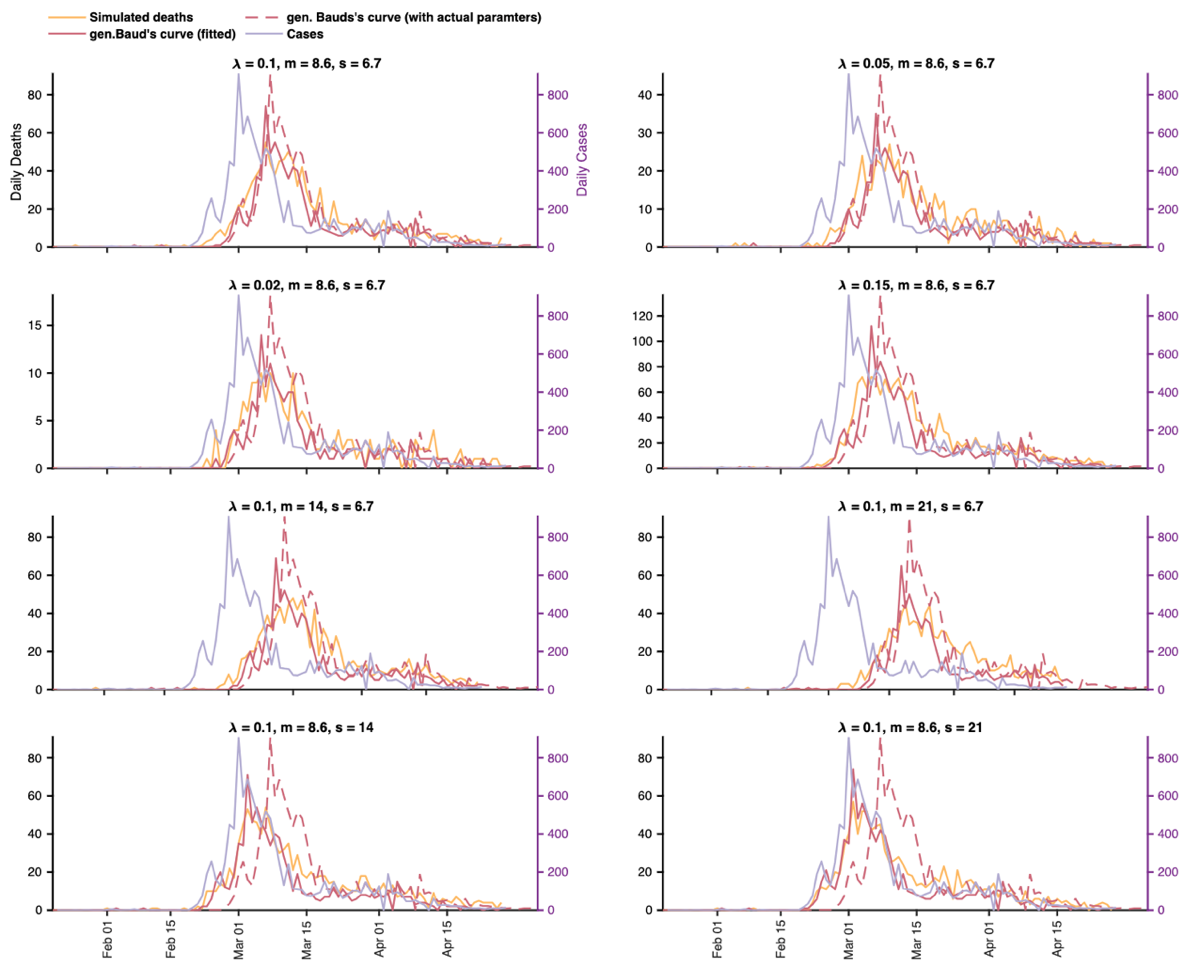


**Figure S 5:** **Why Generalized Baud’s method underestimates the case fatality ratio.** *The figure shows the effect of different case-to-death time parameters on the shape of the estimated fatality curves of Generalized Baud’s method.*

**Figure S 6: Case fatality ratio comparison of the distributed-delay and the direct method.** *Illustration of COVID-19 CFR estimates of the distributed-delay method (green) and direct method (blue) presented on a log scale along the vertical axis. The first point of the distributed-delay CFR curve estimates the reported case to the first reported death. Then, the subsequent CFR estimates are made every ten days, beginning with the first reported death date.*

**Table S 1:** Estimated values of case-to-death times parameters and R^2^

| **S.No** | **Country****/region** | **Mean (m)** | **Standard** **deviation (s)** | **R^2^** |
| --- | --- | --- | --- | --- |
| 1 | Italy | 4.7 | 5 | 0.95 |
| 2 | Spain | 7.2 | 9.6 | 0.75 |
| 3 | Switzerland | 13.2 | 10.4 | 0.75 |
| 4 | Sweden | 7.5 | 3.6 | 0.60 |
| 5 | Germany | 13.4 | 1 | 0.9 |
| 6 | Poland | 6.6 | 3.5 | 0.65 |
| 7 | Netherlands | 6 | 1 | 0.88 |
| 8 | France | 7.4 | 1 | 0.82 |
| 9 | Bulgaria | 26 | 25 | 0.57 |
| 10 | Croatia | 19 | 19 | 0.63 |
| 11 | Cyprus | 9.1 | 0.1 | 0.51 |
| 12 | Czech Republic | 11.9 | 1.1 | 0.74 |
| 13 | Estonia | 11.5 | 0.8 | 0.62 |
| 14 | Greece | 11 | 30 | 0.65 |
| 15 | Hungary | 8,3 | 6.7 | 0.77 |
| 16 | Latvia | 24 | 0.1 | 0.56 |
| 17 | Lithuania | 22.2 | 30 | 0.54 |
| 18 | Luxembourg | 15 | 16.4 | 0.62 |
| 19 | Malta | 3.8 | 0.2 | 0.52 |
| 20 | Portugal | 7.7 | 6.7 | 0.74 |
| 21 | Slovakia | 10.2 | 2.2 | 0.60 |
| 22 | Slovenia | 14.7 | 4.4 | 0.70 |
| 23 | Finland | 17.5 | 0.1 | 0.65 |
| 24 | Denmark | 3.6 | 6.7 | 0.79 |
| 25 | Ireland | 10.9 | 0.16 | 0.65 |
| 26 | Belgium | 6.1 | 2.3 | 0.96 |
| 27 | Austria | 15.9 | 11.2 | 0.75 |
| 28 | Romania | 6.2 | 2.5 | 0.76 |
| 29 | Brazil | 1 | 1.4 | 0.85 |
| 30 | Pakistan | 7 | 30 | 0.91 |
| 31 | India | 12 | 0.1 | 0.7 |
| 32 | South Korea | 23 | 30 | 0.69 |
| 33 | New Zealand | 18 | 0.11 | 0.63 |
| 34 | United States | 6 | 0.79 | 0.83 |

## Computational cost of the distributed-delay method

It is important to note that the method presented in this study requires significant computational power, especially when simulating large-scale scenarios. The computation time of the distributed-delay method depends on several factors, which we have highlighted in Figure S. In our analysis, we utilized COVID-19 data from Italy. As shown in Figure SA, the time required by the method to infer the parameters increases as the number of cases and deaths increases. The computational time is also affected by the swarm-size, a crucial hyperparameter of the PSO algorithm (Figure SB). When the swarm-size is increased, the computational time also increases. As noted earlier, we divided the parametric space of each parameter into $k$ grid-points. The total number of times the method forecasts and fits deaths data to the reported deaths would be of the order $k^{3}$ up until any given day of the outbreak. Therefore, increasing $k$ has an exponential impact on the computation time of the method (Figure SC). Additionally, other factors that may influence the computation time include the bounds of the parameters space in which PSO searches for the minimum. In general, the larger the search space of a parameter, the longer it takes for PSO to find the minimum.

**Figure S7: Influence of different parameters, cases data, and case fatalities data on the computational time of distributed-delay method:** *A) The presented estimates are shown for COVID-19 from Italy from day 80 to 115 at a 5-day interval of the outbreak and by keeping the rest of the parameters as constant. B-C) The estimates for COVID-19 from Italy from day 80 of the outbreak and keeping the rest of the parameters constant.*

# References

1 Linton NM, Kobayashi T, Yang Y, *et al.* Incubation period and other epidemiological characteristics of 2019 novel coronavirus infections with right truncation: a statistical analysis of publicly available case data. *J Clin Med* 2020; **9**: 538.

2 MATLAB:2020. 9.8.0.1359463 (R2020a) Update 1. The MathWorks Inc., 2020.

3 Kennedy J, Eberhart R. Particle swarm optimization. IEEE, 1995: 1942–8.

4 Particle Swarm Optimization. In: Computational Intelligence. John Wiley & Sons, Ltd, 2007: 289–358.

5 Baud D, Qi X, Nielsen-Saines K, Musso D, Pomar L, Favre G. Real estimates of mortality following COVID-19 infection. *Lancet Infect Dis* 2020; **20**: 773.

23/01/2025 18:45:00
